# Intraoperative facial nerve monitoring on operative duration in parotidectomy: a systematic review and meta-analysis

**DOI:** 10.1186/s13005-026-00645-7

**Published:** 2026-07-25

**Authors:** Jheng-Liang Chen, I-Chen Lin, Eric Yi-Liang Shen, Cheng-Ming Luo, Yao-Te Tsai, Shih-Chi Su, Li-Jen Hsin, Yi-Chan Lee

**Affiliations:** 1 Department of Otolaryngology - Head and Neck Surgery, Chang Gung Memorial Hospital, 222, Maijin Rd., Anle Dist., Keelung City, 204 Taiwan; 2 College of Medicine, Chang Gung University, Taoyuan, Taiwan; 3 Department of Radiation Oncology, Chang Gung Memorial Hospital, Taoyuan, Taiwan; 4 Department of Otolaryngology - Head and Neck Surgery, Chang Gung Memorial Hospital, Chiayi, Taiwan; 5 Whole-Genome Research Core Laboratory of Human Diseases, Chang Gung Memorial Hospital, Keelung, Taiwan; 6 Department of Otolaryngology - Head and Neck Surgery, Chang Gung Memorial Hospital, Taoyuan, Taiwan

**Keywords:** Facial nerve monitoring, Parotidectomy, Operative duration

## Abstract

**Background:**

Intraoperative facial nerve monitoring (IONM) can be used during parotidectomy. However, its effect on operative duration remains controversial. This systematic review and meta-analysis aimed to evaluate the association between IONM use and operative duration in parotidectomy.

**Methods:**

A systematic search of MEDLINE, Embase, and the Cochrane Library was conducted for studies published up to January 2026. Studies reporting operative duration for parotidectomy performed with and without IONM were included. Data extraction and quality assessment were performed independently by reviewers. Random-effects meta-analyses were conducted to calculate mean differences (MDs) with corresponding 95% confidence intervals (CIs) for operative duration and tumor size. Subgroup, sensitivity, and publication bias analyses were also performed.

**Results:**

Eight studies comprising 1677 patients were included. Overall, IONM use was associated with a statistically significant reduction in operative duration (MD, − 5.62 min; 95% CI, − 11.02 to − 0.23). In procedure-specific analyses, operative duration was significantly shorter with IONM in superficial parotidectomy (MD, − 9.09 min; 95% CI, − 17.49 to − 0.70), whereas no significant difference was observed in total parotidectomy. Pooled analyses showed no significant differences in tumor size between IONM and non-IONM groups in either the overall cohort or the superficial parotidectomy group.

**Conclusions:**

IONM use is associated with a reduction in operative duration in parotidectomy overall and in superficial parotidectomy. These findings suggest a potential procedural efficiency benefit of IONM in selected parotidectomy cases.

**Supplementary Information:**

The online version contains supplementary material available at https://doi.org/10.1186/s13005-026-00645-7.

## Introduction

Parotid gland tumors account for approximately 2% to 3% of all head and neck neoplasms, and surgical excision is generally the primary treatment modality [1–8]. The concept of intraoperative nerve monitoring (IONM) has evolved substantially since its initial introduction. In 1898, facial nerve (FN) function was assessed through direct observation of facial muscle movement following stimulation [9]. With the clinical introduction of electromyography around the 1980s, nerve monitoring techniques were significantly refined and gradually incorporated into parotid surgery in the early 1990s [10–13]. 

The use of IONM during contemporary parotidectomy varies among surgeons, and the existing literature has primarily focused on its role in preserving FN function. Several reviews have demonstrated that IONM is associated with a reduced incidence of immediate or temporary FN dysfunction following parotidectomy [14–17]. However, evidence regarding its effect on permanent FN paralysis remains inconsistent. Earlier meta-analyses generally reported no significant difference, whereas more recent studies have increasingly suggested a potential protective benefit [14–16]. In contrast to FN functional outcomes, the impact of IONM on operative duration remains underexplored. Some studies suggest that IONM may shorten operative time by facilitating earlier identification of the FN, whereas others have reported no such benefit [18, 19]. Therefore, the present study aimed to evaluate the use of IONM in parotidectomy, with a particular focus on its impact on operative duration.

## Materials and methods

This study was conducted in accordance with the Preferred Reporting Items for Systematic Reviews and Meta-Analyses (PRISMA) guidelines [20]. The primary aim was to assess the effect of IONM on operative duration of parotidectomies.

### Literature search

A comprehensive search was performed across three major electronic databases, including MEDLINE, Embase, and the Cochrane Library. The search covered studies published up to January 2026. Search terms included combinations of “monitoring,” “neuromonitoring,” and “parotidectomy.” In addition, the reference lists of all eligible studies were manually screened to identify additional relevant citations.

### Study selection

Eligible studies included patients undergoing primary parotidectomy and directly compared procedures performed with and without IONM in a two-arm design. Only studies reporting operative duration were included. Studies involving recurrent disease or revision surgery, patients with preoperative FN weakness, or single-arm designs without a comparison group were excluded.

### Data extraction and quality assessment

Data of interest were independently extracted by two reviewers, and any discrepancies were resolved through discussion and consensus. Non-randomized studies were assessed using the ROBINS-I tool, whereas randomized controlled trials were evaluated using the Cochrane Risk of Bias 2 (RoB 2) tool [21, 22]. 

### Variables of interest

The primary outcome assessed in this study was operative duration. Tumor size was evaluated as a secondary outcome.

### Data analysis

Statistical analyses were performed using Comprehensive Meta-Analysis software, version 3 (Biostat, Englewood, NJ, USA), and a random-effects model was applied. Mean differences (MDs) with corresponding 95% confidence intervals (CIs) were calculated for operative duration and tumor size. Statistical heterogeneity was assessed using the *I²* statistic [23]. When a minimum of two studies were available in each subgroup, potential contributors to moderate-to-high heterogeneity were investigated through predefined subgroup analyses (study design, publication year, pathology types, study location, and IONM systems). Sensitivity analyses were performed using a leave-one-out approach to evaluate the robustness of the pooled estimates for the primary outcome. The presence of small-study effects, potentially reflecting publication bias, was evaluated by visual inspection of funnel plots and by Egger’s regression test [24]. The certainty of evidence for the pooled outcomes was assessed using the Grading of Recommendations, Assessment, Development, and Evaluation (GRADE) approach [25]. 

## Results

### Study selection

**Fig. 1:**
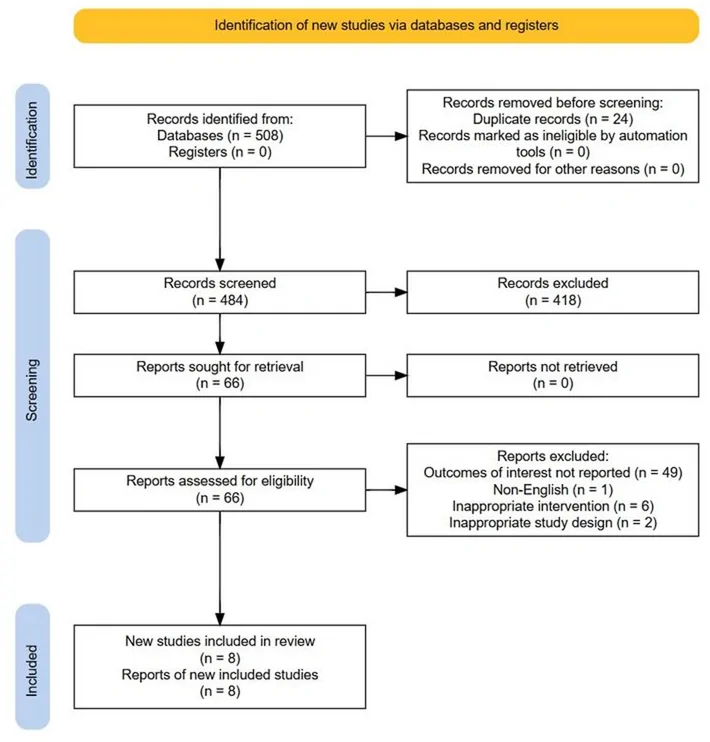
Flowchart of the study selection process

The initial search identified 508 potential publications. After removing 24 duplicates and conducting a preliminary screening of titles and abstracts, the number of studies sought for full-text eligibility assessment was 66 articles. Following a careful review process, a total of 8 studies met all predefined inclusion criteria and were included for the final quantitative synthesis [26–33]. A flowchart illustrating the study selection process is shown in Fig. 1. Detailed search strategies and keywords are provided in the Supplemental Material (eTable 1).

### Characteristics of studies

A total of 1677 patients who underwent primary parotidectomy were included across the eligible studies. Baseline characteristics, such as patient age, sex distribution, and study design, are presented in Table 1. Compliance with the PRISMA reporting standards is documented in the Supplemental Material (eTable 2), and detailed assessments of study quality are provided in the Supplemental Material (eTable 3 and eTable 4).

**Table 1:** Characteristics of the included studies

Author	Year	Country	Study design	Pathology	IONM	Sample size (*n*)		Mean age (years)		Sex (M/F)	
						IONM(+)	IONM(-)	IONM(+)	IONM(-)	IONM(+)	IONM(-)
Terrell et al. [26]	1997	United States	R	B/M	4 Channels	40	40	48.2	47.5	12/28	9/31
Grosheva et al. [27]	2009	Germany	P	B	2 Channels	50	50	52.4*		29/21	23/27
Deneuve et al. [28]	2010	France	R	B/M	2 Channels	46*	41*	46.0*		40/47*	
Savvas et al. [29]	2016	Germany	R	B/M	2 Channels	147	120	52.3	50.6	127/140*	
Graciano et al. [30]	2018	Brazil	RCT	B	4 Channels	52	54	48.5	48.0	17/35	24/30
Kinoshita et al. [31]	2021	Japan	R	B	4 Channels	253	649	53.0†	54.0†	102/151	306/343
Zieliński et al. [32]	2022	Poland	P	B	4 Channels	50	50	54.3	55.8	18/32	16/34
Jirawatthanaanan et al. [33]	2023	Thailand	R	B	4 Channels	15	20	47.3	53.4	11/4	10/10

*M* male, *F* female, *IONM* intraoperative nerve monitoring, *R* retrospective, *P* prospective, *RCT* randomized controlled trial, *B* benign, *M* malignant

* Data presented for the entire group

† Data presented as median values

### Outcomes

#### Operative duration in the overall cohort (minutes)

**Fig. 2:**
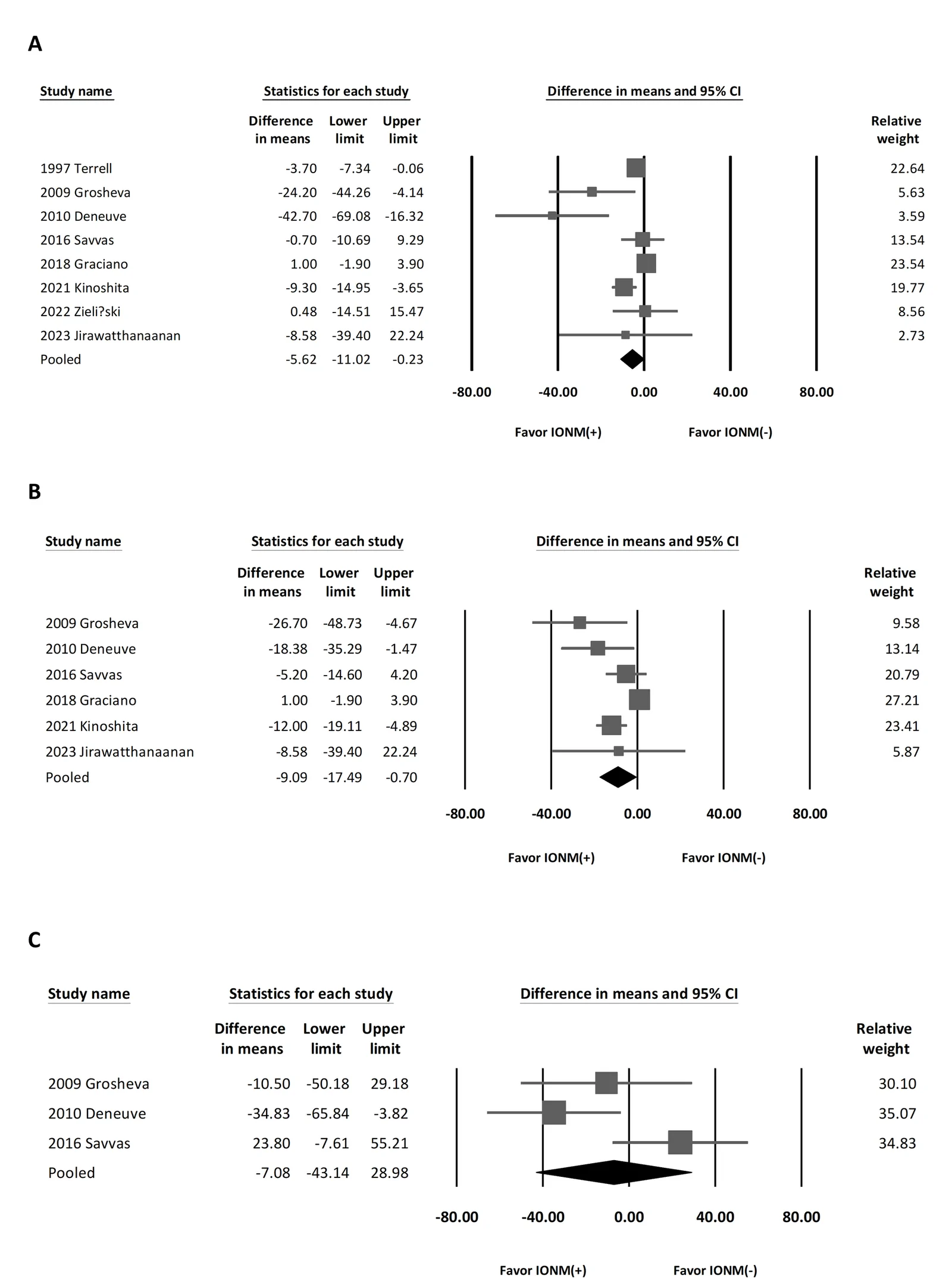
Comparison of tumor size. **A** Overall study population; **B** Superficial parotidectomy group

In a pooled analysis of eight studies that included both superficial and total parotidectomies, the use of IONM was associated with a statistically significant reduction in operative duration (MD, − 5.62; 95% CI, − 11.02 to − 0.23; *I²* = 72.5%) (Fig. 2A).

#### Operative duration in superficial parotidectomy (minutes)

In a pooled analysis of six studies reporting data for superficial parotidectomy, operative duration was significantly shorter in the IONM group compared with procedures performed without IONM (MD, − 9.09; 95% CI, − 17.49 to − 0.70; *I²* = 75.9%)(Fig. 2B).

#### Operative duration in total parotidectomy (minutes)

In a pooled analysis of three studies reporting data for total parotidectomy, no statistically significant difference in operative duration was observed between procedures performed with and without IONM (MD, − 7.08; 95% CI, − 43.14 to 28.98; *I²* = 70.7%) (Fig. 2C).

#### Tumor size in the overall cohort (centimeters)

**Fig. 3:**
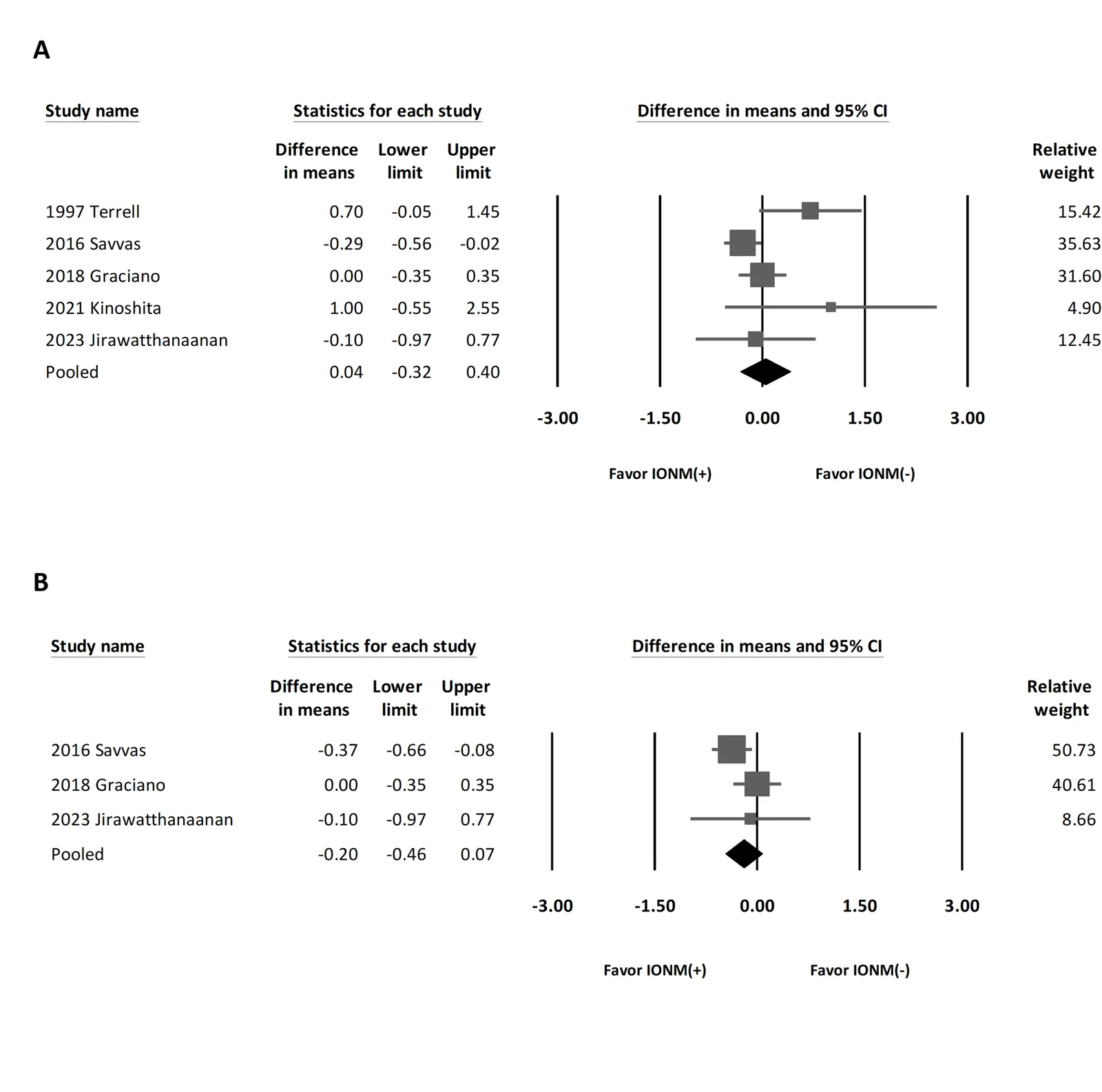
Comparison of tumor size. **A** Overall study population; **B** Superficial parotidectomy group

Tumor size in patients undergoing superficial or total parotidectomy was reported in five studies. No significant difference in mean tumor size was observed between procedures performed with and without IONM (MD, 0.04; 95% CI, − 0.32 to 0.40; *I²* = 53.4%) (Fig. 3A).

#### Tumor size in superficial parotidectomy (centimeters)

Tumor size data for superficial parotidectomy were available in three studies. There was no statistically significant difference in mean tumor size between procedures performed with IONM and those without IONM (MD, − 0.20; 95% CI, − 0.46 to 0.07; *I²* = 24.3%) (Fig. 3B).

### Subgroup analyses

Subgroup analyses of operative duration were performed according to predefined parameters, including study design, year of publication, pathological subtype, study location, and IONM systems. No significant differences between subgroups were observed in either the overall cohort or the superficial parotidectomy group (Supplemental Material, eTables 5 and eTables 6). Owing to the limited number of studies reporting operative duration for total parotidectomy, subgroup analyses for this subgroup were not performed.

### Sensitivity analyses

For operative duration, sensitivity analyses of the overall parotidectomy cohort and the superficial parotidectomy group showed that the direction of the effect estimates remained consistent, although statistical significance was attenuated after exclusion of certain studies. These findings indicate that the pooled estimates may be influenced by individual studies, while the overall trend remained stable. In contrast, the sensitivity analysis for the total parotidectomy group was sensitive to exclusion of the study by Savvas et al., indicating limited robustness. (Supplemental Material, eFigure 1, eFigure 2 and eFigure 3).

### Publication bias

Assessments of publication bias and heterogeneity, including funnel plot analyses and Egger’s regression results, are provided in the Supplemental Material (eTable 7 and eTable 8). The Egger’s test did not indicate the presence of significant publication bias.

### Certainty of evidence assessment

The certainty of evidence assessed using the GRADE approach was low to very low across the five pooled outcomes, mainly because of risk of bias and imprecision .

## Discussion

The present study suggests that the use of IONM during parotidectomy may be associated with a reduction in operative duration overall. Procedure-specific analyses further showed that this reduction was significant in superficial parotidectomy but not observed in total parotidectomy. Pooled analyses of tumor size showed no significant differences between procedures performed with and without IONM in either the overall cohort or the superficial parotidectomy group. To the best of our knowledge, this study represents the first systematic review to specifically evaluate the association between IONM use and operative duration in parotidectomy.

In the surgical management of parotid tumors, both superficial parotidectomy and total parotidectomy are well-established standard procedures [34]. Among the complications associated with these surgeries, FN injury has long been considered a major concern because of its substantial impact on patient quality of life [35, 36]. Efforts to reduce the risk of FN injury have driven the evolution of nerve monitoring techniques over time. In 1898, early nerve monitoring relied on visual observation of facial muscle movement [9]. With the introduction of electromyography around the 1980s, IONM became more reliable and was increasingly adopted [10, 11]. Previous review articles evaluating IONM in parotidectomy have primarily focused on postoperative FN outcomes. Meta-analyses published in 2015, 2021, and 2025 have reported that IONM is associated with a reduced incidence of temporary facial palsy, with increasing evidence also suggesting a potential benefit in reducing permanent facial palsy [14–16]. In contrast, the association between IONM use and operative duration remains controversial and underexplored. Theoretically, IONM may facilitate identification of the FN during surgery and thereby shorten operative time. However, studies addressing this issue have reported conflicting results. The present study therefore aimed to systematically synthesize the available evidence through meta-analysis to address this gap in the existing literature.

The results of our meta-analysis indicate that the use of IONM may be associated with a shorter operative duration in the overall parotidectomy cohort. This effect was also observed in superficial parotidectomy but was not evident in total parotidectomy. One possible explanation is that IONM facilitates early identification of the FN by providing stimulation-evoked electromyography responses [37]. In addition, IONM may assist during dissection of peripheral FN branches, which become thinner and more vulnerable distally [38]. These functions may provide surgeons with additional reassurance and contribute to procedural efficiency [37, 39]. Compared with superficial parotidectomy, total parotidectomy generally requires a longer operative time. As a result, the reduction in operative duration associated with IONM may be less apparent when considered within the overall duration of the procedure. In contrast, because superficial parotidectomy has a shorter baseline operative duration, the impact of operative time reduction associated with IONM may be more noticeable. However, it is crucial to note that the absolute differences in operative duration between the IONM and non-IONM groups were relatively modest in both the overall and superficial parotidectomy cohorts. Given that operative duration can be influenced by various clinical factors, whether this statistically significant time reduction can translate into improvements in operating room efficiency, reduced anesthesia exposure, or cost-effectiveness remains to be explored.

In the present study, most of the included investigations were not randomized trials, making it difficult to ensure that potential confounding variables were balanced between procedures performed with and without IONM. However, tumor size has been reported in prior studies to be associated with operative duration [40–42]. We therefore conducted pooled analyses to determine whether differences in tumor size existed between the IONM and non-IONM groups. The results demonstrated no significant intergroup difference in tumor size in the overall parotidectomy cohort. Similar findings were observed when the analysis was restricted to superficial parotidectomy. Although an analysis of tumor size in total parotidectomy was attempted, the limited availability of data prevented further comparison. While other factors may still influence operative duration, our findings suggest that tumor size was comparable between groups in the present study.

The authors acknowledge several limitations of the present study. First, only eight studies were included, representing a relatively limited evidence base. Second, most included studies were retrospective in design and therefore subject to potential selection bias. Third, heterogeneity in reported outcomes warrants cautious interpretation of the findings. Despite performing subgroup analyses, no specific sources of heterogeneity were identified. Fourth, no significant small-study effects were detected. However, the limited number of included studies reduced the power of funnel plot and Egger’s regression analyses. Therefore, the possibility of publication bias cannot be completely excluded. Fifth, the overall certainty of evidence was limited, underscoring the need for additional high-quality prospective studies. Despite these limitations, we believe that the present study addresses an important knowledge gap and provides clinically relevant insights for practice.

## Conclusion

Our study suggests that the use of IONM is associated with a reduction in operative duration in parotidectomy overall, with a similar effect observed in superficial parotidectomy. In contrast, no significant reduction in operative duration was demonstrated for total parotidectomy. No significant differences in tumor size were observed between monitored and unmonitored groups. Given the limited number of included studies and the sensitivity of some findings to individual studies, the results should be interpreted with caution. Nevertheless, these findings suggest that IONM may provide modest procedural efficiency benefits in selected parotidectomy cases.

## Supplementary Information

Supplementary Material 1.

## Data Availability

All data supporting the findings of this study are available within the paper and its Supplementary Information.
