# Supplementary material for: Intraoperative facial nerve monitoring on operative duration in parotidectomy: a systematic review and meta-analysis
Source: Head Face Med. 2026 Jul 25;22:80. doi: 10.1186/s13005-026-00645-7 (PMC13688902; doi:10.1186/s13005-026-00645-7)
Supplement: Supplementary file 1 [file 13005_2026_645_MOESM1_ESM.docx]

**eTable 1.** Literature Searches and Keywords

- **Medline**

((("monitoring"[Title/Abstract] OR "monitor"[Title/Abstract] OR "neuromonitoring"[Title/Abstract] OR "neuromonitor"[Title/Abstract]))) AND (("parotidectomy"[Title/Abstract] OR "parotid"[Title/Abstract]))

- **Cochrane**

("parotid" OR "parotidectomy") in Title Abstract Keyword AND ("monitor" OR "monitoring" OR "neuromonitor" OR "neuromonitoring") in Title Abstract Keyword AND English in Language NOT "case report" in Publication Type NOT "conference abstract" in Publication Type - (Word variations have been searched)

- **Embase**

parotid AND ('parotidectomy':ti,ab,kw OR 'parotid':ti,ab,kw) AND ('monitoring':ti,ab,kw OR 'monitor':ti,ab,kw OR 'neuromonitoring':ti,ab,kw OR 'neuromonitor':ti,ab,kw) AND 'human'/de AND ('article'/it OR 'article in press'/it OR 'clinical trial'/it OR 'editorial'/it OR 'erratum'/it OR 'letter'/it OR 'short survey'/it OR 'tombstone'/it) AND english:la

**eTable 2.** PRISMA Checklist

| **Section/topic** | **#** | **Checklist item** | **Reported on page #** |
| --- | --- | --- | --- |
| **TITLE** | | |  |
| Title | 1 | Identify the report as a systematic review, meta-analysis, or both. | 1 |
| **ABSTRACT** | | |  |
| Structured summary | 2 | Provide a structured summary including, as applicable: background; objectives; data sources; study eligibility criteria, participants, and interventions; study appraisal and synthesis methods; results; limitations; conclusions and implications of key findings; systematic review registration number. | 2 |
| **INTRODUCTION** | | |  |
| Rationale | 3 | Describe the rationale for the review in the context of what is already known. | 4 |
| Objectives | 4 | Provide an explicit statement of questions being addressed with reference to participants, interventions, comparisons, outcomes, and study design (PICOS). | 4 |
| **METHODS** | | |  |
| Protocol and registration | 5 | Indicate if a review protocol exists, if and where it can be accessed (e.g., Web address), and, if available, provide registration information including registration number. | 6 |
| Eligibility criteria | 6 | Specify study characteristics (e.g., PICOS, length of follow-up) and report characteristics (e.g., years considered, language, publication status) used as criteria for eligibility, giving rationale. | 6 |
| Information sources | 7 | Describe all information sources (e.g., databases with dates of coverage, contact with study authors to identify additional studies) in the search and date last searched. | 6 |
| Search | 8 | Present full electronic search strategy for at least one database, including any limits used, such that it could be repeated. | 6 |
| Study selection | 9 | State the process for selecting studies (i.e., screening, eligibility, included in systematic review, and, if applicable, included in the meta-analysis). | 6 |
| Data collection process | 10 | Describe method of data extraction from reports (e.g., piloted forms, independently, in duplicate) and any processes for obtaining and confirming data from investigators. | 7 |
| Data items | 11 | List and define all variables for which data were sought (e.g., PICOS, funding sources) and any assumptions and simplifications made. | 7 |
| Risk of bias in individual studies | 12 | Describe methods used for assessing risk of bias of individual studies (including specification of whether this was done at the study or outcome level), and how this information is to be used in any data synthesis. | 7 |
| Summary measures | 13 | State the principal summary measures (e.g., risk ratio, difference in means). | 8 |
| Synthesis of results | 14 | Describe the methods of handling data and combining results of studies, if done, including measures of consistency (e.g., I^2^) for each meta-analysis. | 8 |
| Risk of bias across studies | 15 | Specify any assessment of risk of bias that may affect the cumulative evidence (e.g., publication bias, selective reporting within studies). | 8 |
| Additional analyses | 16 | Describe methods of additional analyses (e.g., sensitivity or subgroup analyses, meta-regression), if done, indicating which were pre-specified. | 8 |
| **RESULTS** | | |  |
| Study selection | 17 | Give numbers of studies screened, assessed for eligibility, and included in the review, with reasons for exclusions at each stage, ideally with a flow diagram. | 9 |
| Study characteristics | 18 | For each study, present characteristics for which data were extracted (e.g., study size, PICOS, follow-up period) and provide the citations. | 9 |
| Risk of bias within studies | 19 | Present data on risk of bias of each study and, if available, any outcome level assessment (see item 12). | 9 |
| Results of individual studies | 20 | For all outcomes considered (benefits or harms), present, for each study: (a) simple summary data for each intervention group (b) effect estimates and confidence intervals, ideally with a forest plot. | 10-14 |
| Synthesis of results | 21 | Present results of each meta-analysis done, including confidence intervals and measures of consistency. | 10-14 |
| Risk of bias across studies | 22 | Present results of any assessment of risk of bias across studies (see Item 15). | 9 |
| Additional analysis | 23 | Give results of additional analyses, if done (e.g., sensitivity or subgroup analyses, meta-regression [see Item 16]). | 10-14 |
| **DISCUSSION** | | |  |
| Summary of evidence | 24 | Summarize the main findings including the strength of evidence for each main outcome; consider their relevance to key groups (e.g., healthcare providers, users, and policy makers). | 15 |
| Limitations | 25 | Discuss limitations at study and outcome level (e.g., risk of bias), and at review-level (e.g., incomplete retrieval of identified research, reporting bias). | 19 |
| Conclusions | 26 | Provide a general interpretation of the results in the context of other evidence, and implications for future research. | 20 |
| **FUNDING** | | |  |
| Funding | 27 | Describe sources of funding for the systematic review and other support (e.g., supply of data); role of funders for the systematic review. | Title page |

**eTable 3.** Risk of Bias Assessment of Included Non-randomized Studies (ROBINS-I)

| **Study** | **Confounding** | **Selection of Participants** | **Classification of Intervention** | **Deviations from Intended Intervention** | **Missing Data** | **Outcome Measurement** | **Selection of Reported Results** | **Overall** |
| --- | --- | --- | --- | --- | --- | --- | --- | --- |
| Terrell 1997 | Serious | Moderate | Low | Low | Low | Moderate | Moderate | Serious |
| Grosheva 2009 | Moderate | Low | Low | Low | Low | Low | Moderate | Moderate |
| Deneuve 2010 | Serious | Moderate | Low | Low | Low | Moderate | Moderate | Serious |
| Savvas 2016 | Serious | Serious | Low | Low | Low | Moderate | Moderate | Serious |
| Kinoshita 2021 | Serious | Low | Low | Low | Low | Moderate | Moderate | Serious |
| Zieliński 2022 | Serious | Moderate | Low | Low | Low | Low | Moderate | Serious |
| Jirawatthanaanan 2023 | Serious | Moderate | Low | Low | Low | Moderate | Moderate | Serious |

**eTable 4.** Cochrane Risk of Bias 2 Assessment of the Included Randomized Controlled Trial

| **Study** | **Randomization**  **Process** | **Deviations from**  **Intended Interventions** | **Missing Outcome**  **Data** | **Outcome**  **Measurement** | **Selection of**  **Reported Results** | **Overall Risk**  **of Bias** |
| --- | --- | --- | --- | --- | --- | --- |
| Graciano 2018 | Some concerns | Low | Low | Some concerns | Low | Some concerns |

**eTable 5:** Subgroup analyses of operative duration (overall)

|  | Number of studies | Mean difference | 95% confidence interval | P for subgroup difference |
| --- | --- | --- | --- | --- |
| Study design |  |  |  |  |
| Prospective | 3 | -3.328 | -12.901, 6.245 | 0.49 |
| Retrospective | 5 | -7.592 | -15.062, -0.122 |  |
| Publication year |  |  |  |  |
| > 2010 | 5 | -2.805 | -10.787, 5.176 | 0.12 |
| ≤ 2010 | 3 | -14.082 | -25.646, -2.517 |  |
| Malignancy included |  |  |  |  |
| No | 5 | -5.942 | -14.673, 2.789 | 0.82 |
| Yes | 3 | -7.588 | -18.405, 3.228 |  |
| European studies |  |  |  |  |
| No | 4 | -3.918 | -10.590, 2.755 | 0.35 |
| Yes | 4 | -9.520 | -19.270, 0.231 |  |
| IONM |  |  |  |  |
| 2 channels | 3 | -13.104 | -24.389, -1.820 | 0.14 |
| 4 channels | 5 | -3.409 | -9.519, 2.701 |  |

**eTable 6:** Subgroup analyses of operative duration (superficial parotidectomy)

|  | Number of studies | Mean difference | 95% confidence interval | P for subgroup difference |
| --- | --- | --- | --- | --- |
| Study design |  |  |  |  |
| Prospective | 2 | -4.929 | -16.771, 6.914 | 0.46 |
| Retrospective | 4 | -10.663 | -19.992, -1.333 |  |
| Publication year |  |  |  |  |
| > 2010 | 4 | -5.058 | -12.814, 2.698 | 0.07 |
| ≤ 2010 | 2 | -21.762 | -37.797, -5.727 |  |
| Malignancy included |  |  |  |  |
| No | 6 |  |  | 0.82 |
| Yes | 0 |  |  |  |
| European studies |  |  |  |  |
| No | 3 | -5.401 | -16.714, 5.912 | 0.33 |
| Yes | 3 | -13.959 | -26.799, -1.120 |  |
| IONM |  |  |  |  |
| 2 channels | 3 | -13.959 | -26.799, -1.120 | 0.33 |
| 4 channels | 3 | -5.401 | -16.714, 5.912 |  |

**eTable 7.** Egger's Test Results and Funnel Plots

| **Parameter** | ***I^2^*** | **Egger Test** | **Funnel Plot** |
| --- | --- | --- | --- |
| Operative duration (Overall) | 72.5 | 0.10 |  |
| Operative duration (SP) | 75.9 | 0.05 |  |
| Operative duration (TP) | 70.7 | 0.98 |  |
| Tumor size (Overall) | 53.4 | 0.51 |  |
| Tumor size (SP) | 24.3 | 0.78 |  |

**eTable 8.** GRADE assessment

| **Outcome** | **Risk of bias** | **Inconsistency** | **Indirectness** | **Imprecision** | **Publication bias** | **Other considerations** | **Overall certainty** |
| --- | --- | --- | --- | --- | --- | --- | --- |
| Operative duration (Overall group) | Serious | Not serious | Not serious | Not serious | Undetected | None | ⊕⊕◯◯ Low |
| Operative duration (Superficial parotidectomy) | Serious | Not serious | Not serious | Serious | Undetected | None | ⊕◯◯◯ Very low |
| Operative duration (Total parotidectomy) | Serious | Not serious | Not serious | Serious | Undetected | None | ⊕◯◯◯ Very low |
| Tumor size (Overall group) | Serious | Not serious | Not serious | Not serious | Undetected | None | ⊕⊕◯◯ Low |
| Tumor size (Superficial parotidectomy) | Serious | Not serious | Not serious | Serious | Undetected | None | ⊕◯◯◯ Very low |

**eFigure 1.** Sensitivity analysis of operative duration (overall)

**eFigure 2.** Sensitivity analysis of operative duration (Superficial parotidectomy)

**eFigure 3.** Sensitivity analysis of operative duration (Total parotidectomy)
